# Update on Controls for Isolation and Quantification Methodology of Extracellular Vesicles Derived from Adipose Tissue Mesenchymal Stem Cells

**DOI:** 10.3389/fimmu.2014.00525

**Published:** 2014-10-21

**Authors:** Marcella Franquesa, Martin J. Hoogduijn, Elia Ripoll, Franka Luk, Mahdi Salih, Michiel G. H. Betjes, Juan Torras, Carla C. Baan, Josep M. Grinyó, Ana Maria Merino

**Affiliations:** ^1^Division of Nephrology and Transplantation, Department of Internal Medicine, Erasmus Medical Center, Rotterdam, Netherlands; ^2^Department of Nephrology and Renal Transplantation, Bellvitge Biomedical Research Institute (IDIBELL), Barcelona, Spain; ^3^Department of Nephrology, Bellvitge University Hospital, Barcelona, Spain

**Keywords:** extracellular vesicles, mesenchymal stem cells, differential centrifugation, flow cytometry, electron microscopy, nanosight

## Abstract

The research field on extracellular vesicles (EV) has rapidly expanded in recent years due to the therapeutic potential of EV. Adipose tissue human mesenchymal stem cells (ASC) may be a suitable source for therapeutic EV. A major limitation in the field is the lack of standardization of the challenging techniques to isolate and characterize EV. The aim of our study was to incorporate new controls for the detection and quantification of EV derived from ASC and to analyze the applicability and limitations of the available techniques. ASC were cultured in medium supplemented with 5% of vesicles-free fetal bovine serum. The EV were isolated from conditioned medium by differential centrifugation with size filtration (0.2 μm). As a control, non-conditioned culture medium was used (control medium). To detect EV, electron microscopy, conventional flow cytometry, and western blot were used. The quantification of the EV was by total protein quantification, ExoELISA immunoassay, and Nanosight. Cytokines and growth factors in the EV samples were measured by multiplex bead array kit. The EV were detected by electron microscope. Total protein measurement was not useful to quantify EV as the control medium showed similar protein contents as the EV samples. The ExoELISA kits had technical troubles and it was not possible to quantify the concentration of exosomes in the samples. The use of Nanosight enabled quantification and size determination of the EV. It is, however, not possible to distinguish protein aggregates from EV with this method. The technologies for quantification and characterization of the EV need to be improved. In addition, we detected protein contaminants in the EV samples, which make it difficult to determine the real effect of EV in experimental models. It will be crucial in the future to optimize design novel methods for purification and characterization of EV.

## Introduction

Extracellular vesicles (EV) are lipid bilayer particles coming either from the inside of a cell or formed directly from its cell membrane, and excreted to the extracellular medium. EV participate in cell-to-cell communication by transfer of proteins, bioactive lipids, and nucleic acids (1). The term EV includes exosomes (30–100 nm), microvesicles (100–1000 nm), and apoptotic bodies (50–5,000 nm) (2). Apoptotic bodies are shed from dying cells, while microvesicles are shed from the plasma membrane of viable cells. On the contrary, exosomes are of endocytic origin and are released when multivesicular bodies fuse with the plasma membrane and release their intraluminal vesicles as exosomes to the extracellular milieu (3). Exosomes have been defined based on size, density (1.12–1.19 g/ml), and expression of specific biomarkers (e.g., tetraspanins, annexin, and heat shock proteins) (4). Due to the heterogeneity of the various types of vesicles, it has been suggested to call them collectively EV.

Almost all cell types release EV both under physiological and pathological conditions, such as during cell activation, stress, and apoptosis (5). Recent data indicate that EV have the capacity to modulate immune responses (6) and facilitate tissue regeneration (7). EV are therefore seen as potential therapeutic agents. Adipose tissue-derived mesenchymal stem cells (ASC) may be a suitable source for therapeutic EV. ASC can be easily isolated from adipose tissue obtained by liposuction and expanded manifold *in vitro* (8). Their immunomodulatory properties have proven to be beneficial in the treatment of autoimmune diseases and inhibition of allo-responses after transplantation (9, 10). It has been demonstrated that EV released from ASC may mimic the beneficial effect of ASC treatment (11).

The isolation of EV is a challenging procedure. They have been successfully isolated from supernatant of cell culture and different body fluids (12). The most widely applied method for concentrating and purifying EV is isolation by differential centrifugation. This method consists of a number of centrifugations, which sequentially increase in speed and time, and thus, sequentially pellet smaller particles (12, 13). Although most studies make use of this method for the isolation of EV, vesicle recovery, and contamination cannot be reliably controlled in the separation process because high-speed centrifugation pellets not only EV but also proteins aggregates, lipoprotein particles, and other contaminants (14). Discrepancies within differential centrifugation protocols lead to inconsistencies in the isolated material, and may explain the different biological effects of EV reported by different research groups (15, 16).

In addition to the difficulties of isolating pure EV, progress in EV research is hampered by sub-optimal quantification methods. Due to their small size, EV are below the detection range of conventional quantification methods used for cells. In recent years, various optical and non-optical methods have been developed or adapted for the assessment of EV quantity, size, and features. Further optimization and standardization of these protocols are important for development of the field (17).

In the present study, we isolated EV from ASC conditioned medium by ultracentrifugation and employed multiple methods for the detection and quantification of single vesicles: transmission electron microscopy (TEM), conventional flow cytometry, nanoparticle tracking analysis (NTA), ELISA immunoassay, and total protein quantification.

The aim of our study was to incorporate new controls for the detection and quantification of EV derived from ASC and to analyze the applicability and limitations of the available techniques.

## Methods

### Isolation and culture of human subcutaneous ASC

Subcutaneous adipose tissue from healthy donors that became available as a waste product during the kidney donation procedure was collected after obtaining written informed consent, as approved by the Medical Ethical Committee of the Erasmus University Medical Centre Rotterdam (protocol no. MEC-2006-190). The tissue was collected in minimum essential medium-α (MEM-α) (Sigma-Aldrich, St Louis, MO, USA) supplemented with penicillin (100 IU/ml), streptomycin (100μg/ml) (1% P/S; Lonza, Verviers, Belgium), and 2 mM l-glutamine (Lonza) and stored at 4°C for 3–16 h. ASC were isolated as described previously (18). Cultures were kept at 37°C, 5% CO_2_, and 95% humidity and refreshed twice weekly with MEM-α with 1% P/S, and 15% fetal bovine serum (FBS; Lonza). At 90% confluency, adherent cells were removed from culture flasks by incubation in 0.05% trypsin–EDTA (Life Technologies, Bleiswijk, Netherlands) at 37°C and cells were used for experiments described below or frozen at −150°C until further use. ASC were used for experiments between passages 2 and 5 and their phenotypic markers and osteogenic and adipogenic potential were tested as described before (19). ASC from five donors were used in the experiments. Cell culture supernatants (SN) were processed using MycoAlert^®^kit, according to the manufacturer’s instructions (Lonza), to detect *Mycoplasma* contamination.

### Isolation of EV by differential centrifugation

#### Supernatant collection

Supernatants were collected from conditioned medium of ASC cultures of passage 3–5 at 80% confluence (~1 × 10^6^ cells) 24 h after refreshment with 20 ml of medium (MEM-α containing 5% FBS, 2 mM l-glutamine, 100 U/ml penicillin, and 100 μg/ml streptomycin). Prior to use, the FBS was centrifuged at 100,000 × g for 18 h to remove the possible contaminating EV. In indicated experiments, ASC were cultured in the presence of recombinant human IFN-γ (10 ng/ml) for 3 days, washed with PBS, and cultured with conditioned medium for 24 h. After that, supernatant was collected. Non-conditioned culture medium was used as control (control medium).

#### Isolation of EV

**Figure 1:**
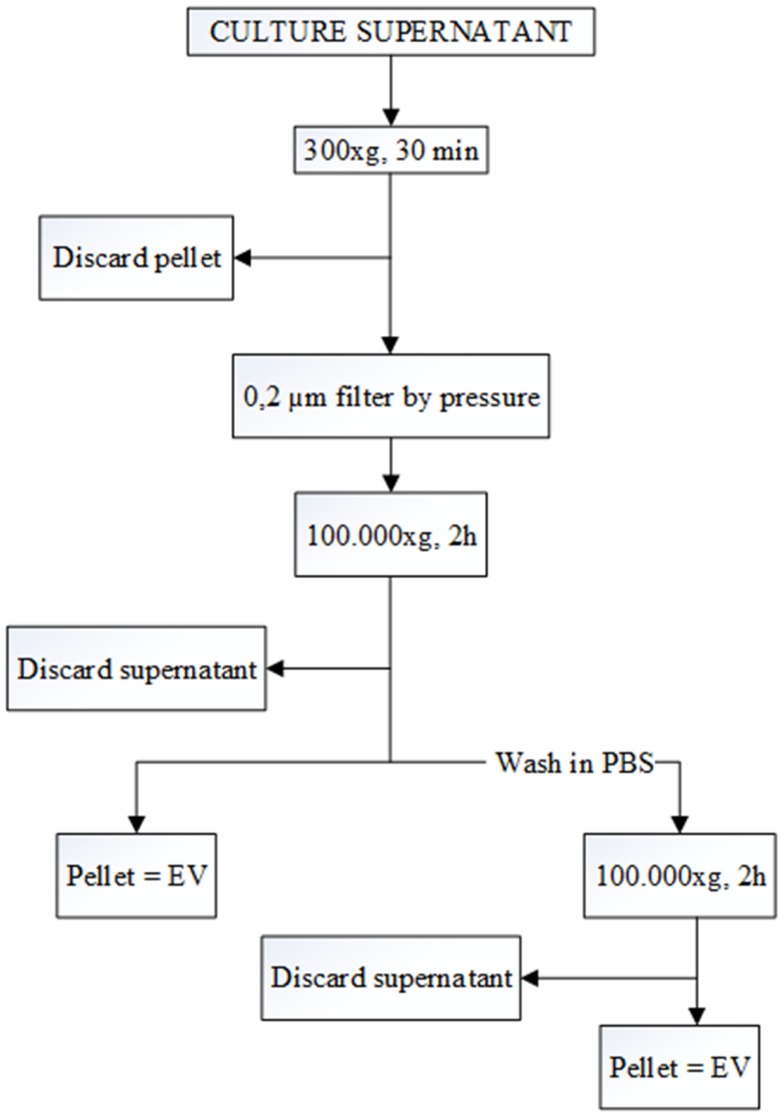
**Flow chart for the EV purification procedure based on differential ultracentrifugation**. The speed and the length of each centrifugation step are indicated at the central line of the arrows. After each the first step, pellet is discarded, and the supernatant is kept for the next step. In contrast, after the two 100,000 × *g* centrifugations, pellets are kept, and the supernatants are discarded.

Differential centrifugation is the most common method used for the isolation of EV from SN (12, 13). First, cells were removed by pelleting by centrifugation at 300 × *g* for 30 min. Subsequently, the supernatant was filtered (pore size 0.2 μm) to remove large particles. Filtered SN were then ultracentrifuged at 100,000 × *g* for 2 h. Some of the pellets were washed with PBS and again pelleted at 100,000 × *g* for 2 h to remove protein contamination (Figure 1). All the samples were ultracentrifuged in polyallomer Quick-Seal centrifuge tubes (25 mm × 89 mm, Beckman Coulter) that have a volume of 39 ml. A Beckman Coulter ultracentrifuge (Beckman Coulter Optima L-90K ultracentrifuge; Beckman Coulter, Fullerton, CA, USA) was used with a fixed angle rotor type 70ti. EVs were collected in 200 μl of filtered PBS.

### Detection methods

#### Electron microscopy

Extracellular vesicles samples used for electron microscopy were re-suspended in PBS and 5 μl was loaded onto formvar carbon-coated grids (Ted Pella Inc., Redding, CA, USA). Samples were fixed in 2% paraformaldehyde and 2.5% glutaraldehyde and contrasted in 2% uranyl acetate. Samples were examined in a DSM 940 A electron microscope (Carl Zeiss NTS, Jena, Germany). Vesicles were measured with the iTEM software (Olympus-SiS, Münster, Germany).

#### Western blot

After ultracentrifugation of control medium and conditioned medium, two fractions (pellets and SN) were collected. The pellets were solubilized (50 μl) and SN were diluted (1:2) in Laemmli buffer. SN were used as technical control. The samples were preheated at 60°C for 15 min. SDS/PAGE was carried out on a 4–20% polyacrylamide gel, and proteins were transferred to Immobilon^®^-P membranes (Millipore, Amsterdam, The Netherlands). The membranes were blocked in 5% milk and were probed overnight at 4°C with CD9 antibody (Santa Cruz biotechnology). After three washes in TBS-Tween 20, membranes were incubated with the secondary antibody (Thermo Scientific, Rockford, IL, USA) for 1 h and washed again. For visualization, blots were exposed to Pierce^®^ enhanced chemiluminescent substrate and measured by Uvitec Alliance 2.7 (Cambridge, UK).

#### Flow cytometric analysis of EV

To obtain fluorescent EV, ASC were labeled with the green fluorescent cell dye PKH-67 according to the manufacturer’s instructions (Sigma). Immediately prior to staining, PKH-67 dye was prepared and added to 1 ml of ASC in suspension (2 × 10^6^ cells). After incubation at 25°C for 10 min, the staining reaction was stopped by adding an equal volume of FBS. Labeled ASC were replated into flaks (seeding density = 1 × 10^6^ cells/175 cm^2^). After 24 h, the cells were attached to the flask, the supernatant was then removed, and the cells were washed with PBS. Conditioned medium was added and collected after 24 h. EV from non-labeled ASC were used as control.

The samples were acquired with a FacsCanto II (Becton Dickinson) and run on the “LOW” setting. The analysis was realized with a SSC/Fluorescent Dot Plot to avoid the error due to FSC detection limit. To compare the detection sensitivity of the flow cytometer, SPHEROTM Nano Yellow Fluorescent Particles (Spherotech, Lake Forest, IL, USA) were used.

### Quantification methods

#### Total protein analysis

Various volumes of cell culture SN were centrifuged (10, 20, 30, 40, and 80 ml) to collect samples with an increasing dose of EV. The number of cells to produce the different amount of volume was ~0.5 × 10^6^, 1 × 10^6^, 1.5 × 10^6^, 2 × 10^6^, and 4 × 10^6^. As control, control medium was used. EV were prepared in two ways. In the first protocol, protein contents of EV were released by protein extraction protocol, isolated EV, or control medium were re-suspended in 200 μl of RIPA buffer (Sigma-Aldrich) with protease inhibitors cocktail (Sigma-Aldrich), and then sonicated for 5 min, three times, vortexing in between. In the second protocol, no protein extraction was performed and the samples were collected in 200 μl of PBS.

Protein contents were measured using a BCA protein assay kit (Thermo Scientific Pierce, Rockford, IL, USA). BSA standard or samples (25 μl) were transferred to a 96 well plate to which 200 μl working reagent was added (working reagent 50:1 ratio of assay reagents A and B). The plate was incubated for 30 min at 37°C, before being analyzed with a spectrophotometer at 562 nm (Victor3, Perkin-Elmer, Waltham, MA, USA).

#### ExoELISA

To generate samples with increasing concentrations of EV, the protocol described in total protein quantification paragraph was performed. Exosome concentrations in the EV samples were determined using commercially available ELISA kits (ExoElisa) purchased from System Biosciences (SBI) (Mountain View, CA, USA). According to the manufacturer’s instructions, after isolation, the EV were diluted in 200 μl of exosome binding buffer and 50 μl of prepared protein standards and EV sample were directly immobilized onto the wells of the microtiter plate. The detection antibody was added to the wells for binding to specific antigen (tetraspanins: CD63, CD9, CD81) protein on the exosomes. A horseradish peroxidase enzyme linked secondary antibody was used for signal amplification. A colorimetric substrate was used for the assay read out. The results were quantified by a microtiter plate reader at 450 nm absorbance and sample readings were extrapolated against a concurrently run standard curve.

#### Nanoparticles tracking analysis

Analysis of absolute size distribution of EV was performed using NanoSight LM10 (NanoSight Ltd., Minton Park, UK). With NTA, particles are automatically tracked and sized based on Brownian motion and the diffusion coefficient. After isolation, the EVs were diluted in 1 ml of filtered PBS. Control medium and filtered PBS were used as controls in this technique. The NTA measurement conditions were temperature 23.75 ± 0.5°C; viscosity 0.91 ± 0.03 cP, frames per second 25, measurement time 60 s. The detection threshold was similar in all the samples (2 multi). Three recordings were performed for each sample.

### Determination of cytokines and growth factors in the EV samples

#### Multiplex bead array kits

To analyze the protein composition of EV samples, a multiplex bead array kit containing EGF, FGF2, G-CSF, IFN-γ, IL-10, IL-12p70, sCD40L, IL-1RA, IL-1b, IL-8, IP-10, MIP1a, and VEGF was used. Conditioned medium (40 ml) from 2 × 10^6^ ASC was collected to perform the EV isolation. EVs were diluted in 200 μl of filtered PBS and 25 μl was added to a 96-wellplate utilizing a custom X-cytokine Milliplex MAP Human Cytokine/Chemokine Magnetic Bead Panel (Millipore Corp., Billerica, MA, USA) following the kit-specific protocols provided by Millipore. Analytes were quantified using a Magpix analytical test instrument, which utilizes xMAP technology (Luminex Corp., Austin, TX, USA), and xPONENT 4.2 software (Luminex). xMAP technology uses fluorescent coded magnetic microspheres coated with analyte-specific capture antibodies to simultaneously measure multiple analytes in a specimen. Concentrations of cytokines (pg/ml) were determined on the basis of the fit of a standard curve for mean fluorescence intensity versus pg/ml.

## Results

### ASC characterization

**Figure 2:**
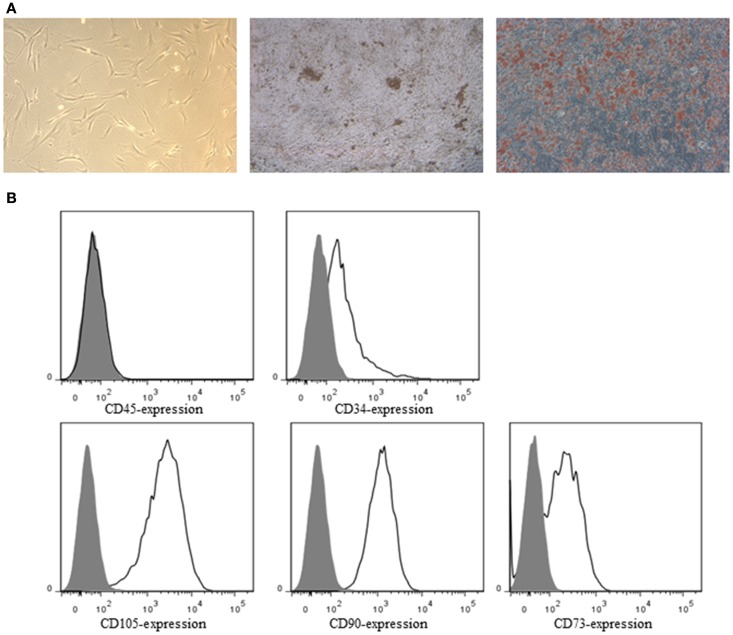
**(A)** Bright-field image of ASC in culture (left), osteogenic (middle), and adipogenic (right) differentiation of ASC. ASC were stained by von Kossa staining for calcified nodules (black) and by oil red O for lipid-filled vesicles (red), respectively. **(B)** ASC were characterized by flow cytometry. ASC were negative for CD34 and CD45, and positive for the ASC markers CD73, CD90, and CD105. Closed histograms: unstained cells, open histograms: stained cells.

ASC demonstrated a spindle-shaped morphology in culture. When cultured under the appropriate conditions the cells were capable of differentiating into the osteoblastic and adipogenic lineages, characterized by the deposition of calcified nodules and production of lipid-filled vesicles, respectively (Figure 2A). Flow cytometric analysis demonstrated ASC to be negative for CD34 and CD45, but positive for the ASC markers CD73, CD90, and CD105 (Figure 2B). All the SN were negative for *Mycoplasma*.

### Detection of EV in conditioned medium and control medium

#### Electron microscopy and western blot

**Figure 3:**
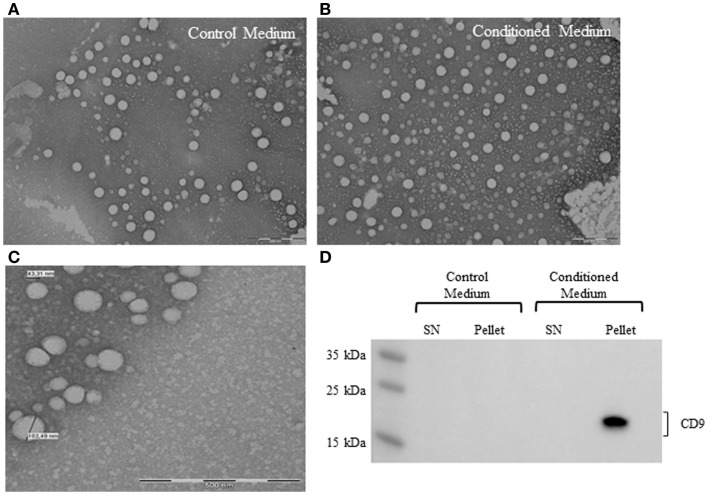
**Electron micrographs analyses of EV from control medium (A) and conditioned medium (B)**. Structural analysis of ASC-derived EV shows the presence of vesicles in the size range of 40–120 nm. Scale bar 500 nm **(C)**. Representative western blot analysis of exosome marker (CD9) in control medium and conditioned medium **(D)**.

To confirm the presence of EV derived from ASC in conditioned medium, samples were processed for electron microscopy. Control medium was used to determine vesicle contamination from the vesicle-depleted serum. The analysis showed the presence of nano-sized vesicles in both conditioned medium and control medium (Figures 3A,B). EV had a rounded shaped, and the diameter was between 40–120 nm, based on the smallest and greatest observed vesicles in several micrographs (Figure 3C). The distribution of the EV in the sample was not homogeneous, some empty fields, and others with aggregated EV were observed.

Western blot analysis revealed the presence of human CD9 marker in the conditioned medium pellet (Figure 3D). Control medium was negative for the expression of human CD9, suggesting that the EV detected in the medium were FBS origin.

#### Flow cytometry

**Figure 4:**
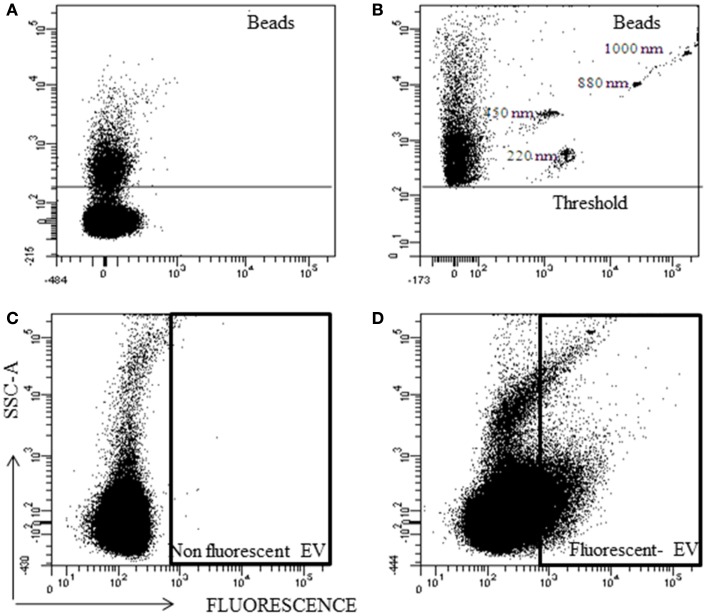
**Representative analysis of nano-fluorescent beads and EV by flow cytometry**. Scatter (SSC) and fluorescent resolution of nanobeads with various sizes **(A)** without SSC threshold, and **(B)** with SSC threshold. **(C)** Non-fluorescent EV and **(D)** fluorescent EV plot.

Fluorescent nanobeads were acquired without SSC-A threshold and due to the noise, the nanobeads could not be detected (Figure 4A). Using a SSC threshold of 200 arbitrary units, the lower sensitivity of the instrument was established, and a part of the noise was eliminated. With this configuration, the nanobeads were detected (Figure 4B). But, fluorescent particles below the threshold cannot be analyzed.

Extracellular vesicles without fluorescence were used as negative control (Figure 4C) and with fluorescence to know their location in the SSC/Fluorescent dot plot (Figure 4D). The majority (>90%) of the fluorescent EVs were at noise area, and resolving the noise from Fluorescent EV was not possible.

### Quantification of EV in supernatants

#### Protein quantification

One of the main methods of quantifying the number of EV is to determine the protein contents of EV. To do this, we isolated EV from increasing volumes of conditioned medium and control medium. The pellets were prepared with and without protein extraction.

**Figure 5:**
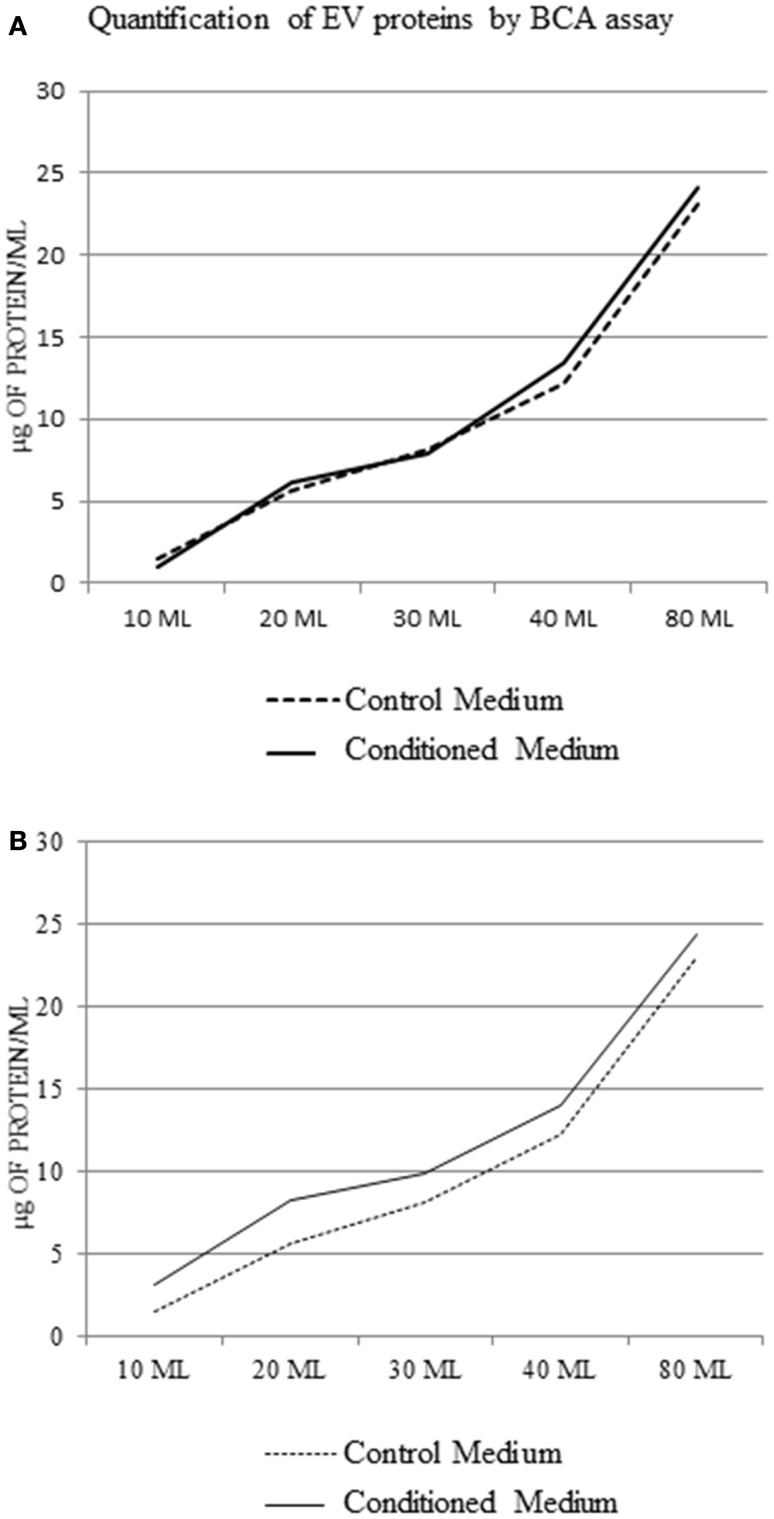
**Extracellular vesicle protein concentration in the control medium and conditioned medium; BCA assay using a protocol without extraction of EV protein (A), and with protein extraction (B)**.

Analyzing the protein concentration in the pellets without protein extraction, we observed that there was no difference between control medium and EV samples (Figure 5A). After protein extraction, the difference between pellet from conditioned medium and control medium at each concentration point was not significant (1.5 ± 0.9 μg/ml protein) (Figure 5B).

### ExoELISA

To quantify the exosome concentration in the EV samples, three ELISA kits were used to detect the tetraspanin proteins CD9, CD81, and CD63.We encountered some technical problems with the kits. The main issue was that the standard protein of the kits produces a low-intensity signal. The absorbance at 450 nm of the maximum concentration of the standard protein was 0.2 ± 0.1, whereas the expected values should be ~1.5. In the CD63 and CD81 ELISA, our samples showed a signal higher than the standard curve but the results, at duplicated wells, had a large SD.

### Nanoparticles tracking analysis

**Figure 6:**
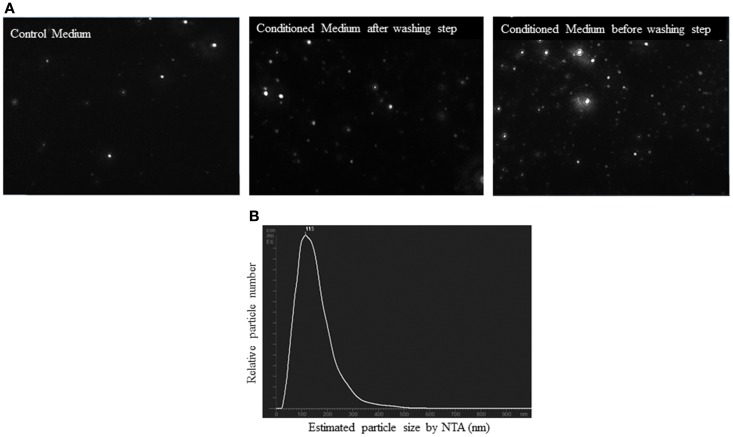
**Nanoparticle tracking analysis (NTA) of conditioned medium from ASC culture**. **(A)** Screen shots from NTA videos of control medium, conditioned medium, and conditioned medium before washing step in PBS. **(B)** Size distribution graph of EV from ASC with a peak at 115 nm.

Before analysis of the samples by NTA, we checked that salts aggregates from the PBS did not produce any background and the equipment was free of any contaminant particles from other users. Control medium showed a considerable number of particles (2.5 × 10^8^ ± 0.5 × 10^8^ particles/ml). However, the concentration of the EV from conditioned medium was higher than control medium (5 × 10^8^ ± 0.4 × 10^8^ particles/ml, *p* < 0.05). It was observed that before the washing step, conditioned medium showed a higher number of particles (28.5 × 10^8^ ± 3 × 10^8^ particles/ml) than after this step (*p* < 0.01). Figure 6A is a representative screen shot of the NTA videos for control medium, conditioned medium after/before washing step. With respect to the size, the EV population was homogenous. NTA estimated the size of the EV between 90 and 150 nm (Figure 6B).

### Detection of cytokines in EV samples

**Figure 7:**
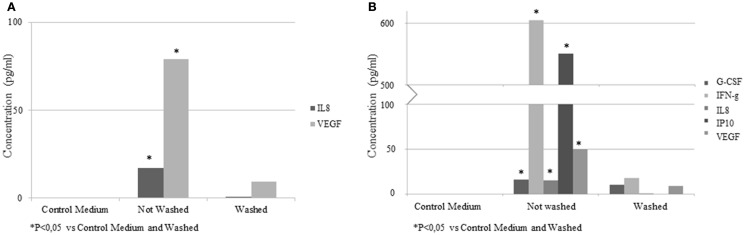
**Concentrations of cytokines and growth factors quantified by multiplex bead array kit in EV samples from control medium and conditioned medium**. Results are expressed as pg/ml. **(A)** EV from ASC; **(B)** EV from IFN-γ pretreated ASC.

To determine the cytokines or growth factor present in the EV samples, a multiplex bead array kit was used. IL-8 (16.9 ± 7.1 pg/ml) and VEGF (78.8 ± 21.4 pg/ml) were detected in the samples (Figure 7A). In an additional experiment, EVs were isolated from conditioned medium of ASC stimulated with IFN-γ. Elevated concentrations of G-CSF (16.1 ± 2.4 pg/ml), IFN-γ (604.8 ± 112.3 pg/ml), IL-8 (15 ± 1.7 pg/ml), IP-10 (550.7 ± 98.4 pg/ml), and VEGF (50 ± 7.1 pg/ml) were found (Figure 7B). In the control medium, no factors were detected. The concentrations of these factors were significantly lower when the EVs were washed with PBS. No significant levels of EGF, FGF2, IL-10, IL-12p70, sCD40L, IL-1RA, IL-1b, and MIP1a were detected.

## Discussion

In the present study, EV were isolated from ASC and their purity and quantity determined with currently available techniques. The limitations of these methods have been summarized in Table 1.

**Table 1:** **Summary of main characteristics of the EV detection and quantification methods**.

	Characteristics
**DETECTION METHODS**	
Electron microscopy	Direct evidence for the presence of EV
	Assessments of morphology and size
	No quantification of EV
	Need an expert in electron microscopy
Flow cytometry	Detection of EV bigger than 300 nm
	Low detection threshold (only analysis of large EV)
Western blot	Detection of specific EV subset
**QUANTIFICATION METHODS**	
BCA protein assay	Easy protocol
	Low-cost method
	No specific information about EV concentration
ExoELISA (SBI)	Specific for exosome proteins
	Technical troubles. Unreliable
NTA	Analysis of absolute concentration of particles
	Assessment of the particles size
	No distinguishment of EV from aggregated protein

Working with EV requires healthy cell cultures. One of the most important requirements for working with EV is to verify that the cell culture is *Mycoplasma* free. We used a colorimetric test to demonstrate that our ASC culture had no *Mycoplasma* contamination. Several authors showed a potential immunosuppressive mechanism of mycoplasmas-infected cells through the release of vesicles (20). They reported that a few dendritic cells, which produced EV fractions with potent capacity to stimulate B cell, but not T cell proliferation, contained *Mycoplasma* isolated along with vesicles (21). Despite the importance of detecting *Mycoplasma* contamination in studies about the immunomodulatory capacity of the EV, this is not standard practice.

The first challenge of working with EV is the isolation of these particles. Many different procedures to obtain EV have been described in the literature (22) and they potentially obtain EV with different properties and compositions (23). Differential centrifugation is the most common method to purify EV from body fluids and conditioned medium. Several protocols are available, generally it consists of multiple steps: first a low speed spin, followed by high-speed spin at 100,000 × *g*. To obtain more purified EV and eliminate protein contaminations, the pellet is washed again in a large volume of PBS and centrifuge an additional time at 100,000 × *g*. The EV isolation from ASC presents the inconvenience that it is necessary to use serum for the cell culture. Several authors have demonstrated that serum deprivation produces changes in the expression pattern of markers in ASC (24) and in the secretome (25). To avoid contamination with EV from the serum, it has been described that serum should to be ultracentrifuged before added to ASC culture. But, we also suggest that non-conditioned medium (control medium) needs to be prepared with the same protocol as the conditioned medium. With this control, the possible background of the medium in the detection and quantification techniques could be detected. In order to confirm the presence of EV, conditioned medium, and control medium were analyzed by electron microscopy. Both preparations contained a population of EV with a size range between 40 and 150 nm. This technique provides direct evidence for the presence of EV, but it is of limited use for concentration measurements. The analysis of the western blot confirmed the presence of CD9 positive exosomes in the samples, but with this method, it is not possible to obtain global information about all the types of EV contained in the samples. It would be necessary, a common marker for exosomes and microvesicles, because both precipitate with the differential centrifugation protocol (13).

Flow cytometry could be a powerful method for qualitative and quantitative characterization of EV. The EV are usually labeled with fluorescent membrane intercalating dye PKH-67 to be detected by flow cytometry. For removal of excess of PKH-67, EVs are subjected to gradient centrifugation on sucrose (26). We decided to not use this technique because only EV with a particular density but not the total population of EV can be obtained by gradient centrifugation on sucrose. This subpopulation would differ from the EV isolated with the differential centrifugation protocol. To resolve this technical issue, ASC were labeled with PKH-67, so that secreted EV would be PKH-67 positive (27). The fluorescent EV were isolated by differential centrifugation and analyzed in a conventional BD FacsCanto II with a fluorescent threshold. Positive fluorescent particles were shown in a SSC/Fluorescent plot. In our experimental conditions, the fluorescence signal was overlapping with the noise, making conventional flow cytometry unsuitable for the quantification and characterization of EV.

Various methods have been developed or adapted for the assessment of EV quantity (17). Optimization and standardization of protocols remain an important task. One of the most used techniques to quantify the EV is the quantification of the total proteins of the EV with BCA protein assay. Two protocols for the quantification of the EV are available in the literature, one with EV protein extraction (14), and one without (12). However, protein measurements of EV-containing pellets are inadequate to quantify EV as pellets from a high-speed spin contain proteins complexes/aggregates, lipoprotein particles, and other contaminants.

ELISA for tetraspanin proteins has the advantage of measuring the concentration of EV specific protein as it is based on EV specific antigen–antibody immune reaction. This reaction would confer specificity to measurement of the EV. None of the currently used protocols of EV purification are likely to separate the different types of vesicles (13) and there is not a common marker for all the EV (28). Consequently, we could only analyze some vesicle subsets like exosomes, because they express similar membrane proteins (tetraspanins) (29), but it is not possible to know the total concentration of the EV in our samples with the ELISA. Additionally, the commercial ExoELISA that is available for measuring exosome concentrations showed several technical troubles.

Nanoparticle tracking analysis is an optical particles tracking method for obtaining concentration and size distribution of EV populations and is capable of detecting single particles with a diameter as low as 50 nm (2). We quantified control medium and conditioned medium before and after washing step with NTA, to know whether this step of the protocol reduced the yield of EV. Some contaminant particles in control medium were observed, most likely coming from the serum. This contamination could alter the measured concentration of the samples. Respect to the washing step, a loss of particles was observed after this step but, with NTA, it is not possible to determine whether the loss of particles during the washing step represents the loss of protein aggregates or EV (30).

Due to the difficulty to obtain EV without protein contamination by differential centrifugation (16), we decided to use a multiplex bead array kit to analyze the presence of a range of cytokines and growth factor produced by the ASC in EV samples. Factors like VEGF and IL-8 were detected. ASC stimulated with IFN-γ secreted more factors (IP-10, IFN-γ, G-CSF) than ASC under normal conditions (31) and these factors were also found in the EV samples. The washing step decreased the concentration of these factors but did not eliminate them completely. These factors were not present in the control medium indicating that they were released by ASC. We can speculate about the position of these factors, they could be shuttled in the membrane of the EV or they could be contaminant factors that precipitate with the EV. Due to that these factors can be detected in ASC SN, without differential centrifugation protocol, with a concentration higher than in the EV samples, it is more likely to be contaminating proteins from the ASC.

## Conclusion

The current technologies for isolation, quantification, and characterization of EV need to be improved. The control medium showed the background that is produced by the medium in the detection and quantification techniques and even could be necessary to determine the effects that the vesicles from the serum could have in functional assays. Measurement of soluble factors in EV samples should be incorporated in the studies to obtain reliable results about the effects of the EV.

## Conflict of Interest Statement

The authors declare that the research was conducted in the absence of any commercial or financial relationships that could be construed as a potential conflict of interest.
